# Items

**Published:** 1903-05

**Authors:** 


### ITEMS.

---

To the American Medical Association.—The Queen and Crescent Route offers (see adv. page xxxvii.) to transport visitors to New Orleans on account of the meeting of the American Medical Association from Buffalo and return, for one fare. This is in accordance with the liberal arrangements of the Southern Passenger Association and should induce many physicians and their families to attend. It is not often in a lifetime that one can go so far and see so much for so little money.

---

Some of our readers no doubt will be interested to learn that the Oppenheimer Institute, in addition to its place in New York and branches in Philadelphia, Detroit and Pittsburg, has opened a branch at No. 2901 Pacific Avenue, Atlantic City, N. J. Physicians will doubtless prefer to have a considerable number of their patients go to Atlantic City for treatment, as they will there have the benefit of a most desirable climate and attractive and restful surroundings.

---

Merit and Reliability with Consequent Success.— The Antikamnia Chemical Company, for many years located at No. 1723 Olive Street, St. Louis, Mo., has moved into its new home, Nos. 1622, 1624, 1626 Pine Street, in that city. The new laboratory is fully equipped with all the latest chemical appliances and machinery, which afford increased and needed capacity for the manufacture of the well known and reliable antikamnia preparations. The company states that its sales during 1902 were the largest in the history of its business, and that the demand for its products is constantly growing, is demonstrated by the fact that the first three months of this year show a pronounced increase of sales over those of the corresponding months of last year. It is this growth of the business which necessitated the removal into larger quarters, where the company has 75 per cent. more space than in its old plant. This is a most satisfactory showing and indicates the prosperity resultant from merit and integrity applied to vigorous business principles.

An increase of the medical corps of the navy to the number of 150 was provided for by the last congress, 25 of which are to be appointed each calendar year for six years. Appointments are to be made in the grade of assistant surgeon from well qualified physicians, citizens of the United States between the ages of 21 and 30 years. Examining boards are in continuous session at Washington, D. C., and Mare Island, Cal. Applications should be made to the secretary of the navy, accompanied by testimonials as to moral standing and citizenship, no political influence being required.

---

Fairchild Bros. & Foster have obtained another conviction for substitution, the guilty party in this instance being Walter L. Conwell, of Boston. The final decree in the case says:

That the defendant, Walter L. Conwell, his agents, servants and employes be, and they hereby are, perpetually enjoined from selling or dispensing either at the drug store of the said Walter L. Conwell, at No. 210 Shawmut Avenue, in the City of Boston, or elsewhere, any "Essence of Pepsine" or pharmaceutical preparation of any sort or kind whatsoever not manufactured by Fairchild Bros. & Foster, in imitation of or in substitution for "Fairchild's Essence of Pepsine" whenever "Fairchild's Essence of Pepsine" is prescribed or asked for, and that they be, and hereby are, perpetually enjoined from representing by any word or action that any preparation sold by the said defendant not manufactured by Fairchild Bros. & Foster is "Fairchild's Essence of Pepsine."

It is further ordered that a writ of injunction restraining the defendant as above set forth issue forthwith.

In this state a bill has just passed the legislature and only awaits the governor's signature to become a law, which will adequately punish such villainous procedures as resulted in the foregoing decree.

---

### DEATH OF HON. JAMES O. PUTNAM.

After the personal item, on page 770, relating to Mr. Putnam was printed, and just as this page enters the press, we learn that he died about 9 o'clock this (Friday) morning, April 24, 1903. Mr. Putnam was a member of the council from the foundation and for many years chancellor of the University of Buffalo, hence his death will be sadly felt by many physicians besides a whole community of citizens in general.

